# Frequency and Severity of Diabetic Retinopathy in Patients With Diabetic Foot Ulcers at a Tertiary Hospital in Bangladesh

**DOI:** 10.7759/cureus.107963

**Published:** 2026-04-29

**Authors:** Ametav Das, Debabbrata Paul, Md Shafiqul Islam, Leiby Orellana Banegas, Abdulaziz Ahmed Salem Al-khulaifi, Md Rezwanul Hasan, Habiba Sultana, Md Sajidul Huq

**Affiliations:** 1 Cornea Department, Deep Eye Care Foundation, Rangpur, BGD; 2 Ophthalmology Department, Sher-E-Bangla Medical College, Barishal, BGD; 3 Medicine Department, Universidad Catolica de Honduras, San Pedro Sula, HND; 4 Medicine Department, Mendero Medical Center, Cebu, PHL; 5 Vitreo-Retina Department, Deep Eye Care Foundation, Rangpur, BGD; 6 Glaucoma Department, Deep Eye Care Foundation, Rangpur, BGD; 7 Public Health Department, Deep Eye Care Foundation, Rangpur, BGD

**Keywords:** diabetic foot ulcer (dfu), diabetic retinopathy, prevalence rate, type 2 diabetes mellitus (t2d), wagner's classification

## Abstract

Background

Diabetic retinopathy (DR) and diabetic foot ulcers (DFUs) are major complications of type 2 diabetes mellitus (T2DM) that significantly contribute to morbidity, particularly in low- and middle-income countries such as Bangladesh. While DR is a leading cause of vision loss, DFU is a major contributor to lower-limb amputation. These complications may coexist as manifestations of systemic vascular damage. This study aimed to determine the frequency and severity of DR among patients with DFU and to evaluate its association with ulcer severity using Wagner’s classification.

Methodology

This cross-sectional study was conducted from August 2021 to July 2022 at Sher-E-Bangla Medical College Hospital, Barishal, Bangladesh. A total of 100 adult patients with T2DM and Wagner-graded DFU were included using purposive sampling. Ophthalmologic evaluation included visual acuity assessment, slit-lamp biomicroscopy, dilated fundus examination, fundus photography, and optical coherence tomography. DR was graded according to the Early Treatment Diabetic Retinopathy Study (ETDRS) classification, and DFU severity was assessed using the Wagner grading system. Laboratory parameters, including HbA1c, lipid profile, serum creatinine, and hemoglobin, were recorded. Statistical analysis was performed using SPSS version 23.0, with chi-square tests applied to assess associations (p < 0.05 considered significant).

Results

The mean age of participants was 55.7 ± 10.9 years, and 67 (67%) patients were male. DR was present in 80 (80%) patients, with mild non-proliferative diabetic retinopathy (NPDR) being the most common subtype in 36 (36%) patients, followed by severe NPDR in 21 (21%) patients, moderate NPDR in 14 (14%) patients, proliferative diabetic retinopathy (PDR) in 9 (9%) patients, high-risk PDR in 3 (3%) patients, and advanced diabetic eye disease in 6 (6%) patients. Visual impairment (≤6/60) was observed in 33 (33%) patients in the right eye and 36 (36%) patients in the left eye. A significant association was found between DR severity and higher Wagner grades of DFU (p = 0.001). Patients with DR had a longer duration of diabetes (15.3 ± 6.6 vs. 9.7 ± 4.5 years, p = 0.006), were more likely to smoke (31 patients, 38.8% vs. 0 patients, 0%; p = 0.018), and had lower hemoglobin levels (10.9 ± 1.8 vs. 12.1 ± 1.1 g/dL, p = 0.046).

Conclusion

DR is highly prevalent among patients with DFUs, with severity closely associated with ulcer grade. These findings support the need for routine ophthalmologic screening in patients presenting with DFU and highlight the importance of integrated care to reduce the risk of both vision loss and limb amputation.

## Introduction

Diabetes mellitus (DM), particularly type 2 diabetes mellitus (T2DM), is a chronic metabolic disorder characterized by hyperglycemia due to insulin resistance and comparative insulin deficiency [1]. It is estimated that 537 million adults (20-79 years) globally were living with diabetes in 2021, a figure that the International Diabetes Federation projects will rise to 783 million by 2045 [2]. T2DM accounts for 90%-95% of all diabetes cases, with rapid, concerning increases in low- and middle-income countries (LMICs) due to urbanization and lifestyle changes [3]. In the South-East Asian country of Bangladesh, which is a lower-middle-income nation, the prevalence of diabetes has increased dramatically to 13.2% in adults, the second highest in South-East Asia [4].

Diabetes is strongly associated with both microvascular and macrovascular complications such as retinopathy, nephropathy, and neuropathy [5], and ischemic heart disease, peripheral vascular disease, and cerebrovascular disease [6], which cause organ and tissue destruction in one-third to half of individuals with diabetes [7]. Screening and early diagnosis of diabetes, followed by immediate treatment of glycemia and cardiovascular risk factors, are probable to have significant health benefits. In Bangladesh, diabetes management is critically poor, with only 30.9% of individuals aware of their condition and a subsequent 69.1% remaining undiagnosed, while treatment and control rates stand at just 28.2% and 26.3%, respectively, reflecting substantial delays in intervention [8].

The most common microvascular complication in diabetes mellitus is diabetic retinopathy (DR), which is the leading cause of blindness in the adult working age group [9]. The spectrum of DR progresses from early non-proliferative changes, such as microaneurysms and retinal hemorrhages, to vision-threatening (PDR) and diabetic macular edema [10]. In 2020, the lobal prevalence of DR was estimated at 22.3%, affecting approximately 103 million individuals, a figure that is projected to exceed 160 million by the year 2045 [11]. The risk increases with longer duration (>10 years), suboptimal glycemic control (HbA1c >7%), hypertension, dyslipidemia, and nephropathy [12,13]. In contrast, diabetic foot ulcers (DFUs) are macrovascular/neuropathic sequelae and affect 19%-34% of diabetics in their lifetime, with an annual prevalence of 6.3%, which is higher in T2DM (6.4%) than type 1 DM (5.5%) [14,15]. Peripheral neuropathy, ischemia, and infection are all features of DFU pathogenesis, contributing to 85% of non-traumatic amputations, affecting up to one-third of diabetics [16]. Co-existing complications such as DR and nephropathy are the most important risk factors since they slow the healing of the ulcer, fail to ensure infection control, and increase the incidence of amputation [17].

Multiple studies have demonstrated a strong association between DR and DFUs, with the occurrence of one complication greatly heightening the risks of the other [18]. Meta-analyses and observational data show that DR is highly elevated in patients with DFU, usually 80%-96%, and a significant number of patients have proliferative or severe forms, and DR has a significant independent predictive value of increased DFU incidence, severity, and adverse outcomes including amputation [19-21]. The co-occurrence of these sight- and limb-threatening conditions highlights a major gap in daily multidisciplinary assessment in South Asian settings, such as Bangladesh, where the management of diabetes is still suboptimal, and the complications are worsened by the delay in their diagnosis [22,23]. Therefore, the aim of this study was to determine the frequency and severity of DR in patients with DFU at a tertiary hospital in Bangladesh and to explore the relationship between DR and DFU grades using Wagner's classification.

## Materials and methods

Study design and setting

**Figure 1:**
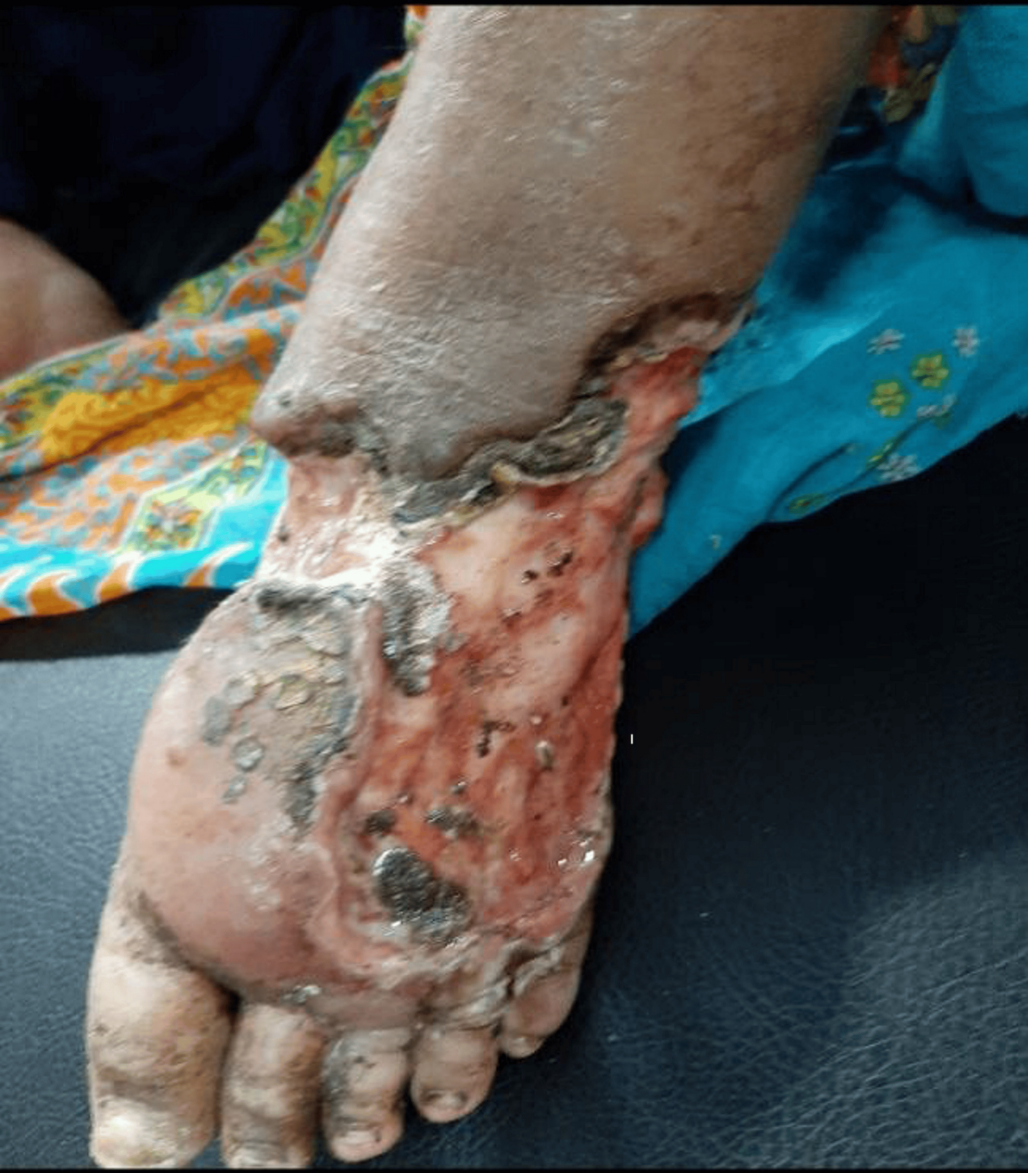
Patient with diabetic foot ulcer

This cross-sectional study was conducted at the Department of Ophthalmology in collaboration with the Department of General Surgery at Sher-E-Bangla Medical College Hospital, Barishal, Bangladesh. Data collection spanned from August 2021 to July 2022. The study population comprised male and female patients with tT2DM who presented with DFUs (Figure 1) at either the outpatient department or the indoor general surgery ward during this period.

Sample size and sampling technique

The initial sample size was calculated using the standard formula for cross-sectional studies: n = z²pq/d², where z = 1.96 at 95% confidence interval, p = 90% (based on previous prevalence), q = 1-p, and d = 5% allowable error. The calculated sample size was 138 participants. However, due to time constraints and the challenges posed by the COVID-19 pandemic, a total of 100 patients were eventually enrolled. Participants were selected using purposive sampling.

Inclusion and exclusion criteria

Patients aged 18 years or older with diagnosed tT2DM and active DFUs classified according to the Wagner grading system were included. We excluded patients with dense cataracts that precluded adequate fundal examination, those under 18 years of age, and those whose foot ulcers resulted from Buerger's disease rather than diabetic complications.

Study procedures

Eligible patients were approached during their hospital visits, and the purpose and potential benefits of the study were explained thoroughly in simple, accessible Bengali. Written informed consent was obtained from each participant before enrollment. A detailed medical history was taken, followed by a comprehensive physical examination, and all findings were recorded systematically.

Ocular examinations were performed by a trained ophthalmologist. Distance visual acuity was assessed using Snellen's chart positioned at 6 meters. For patients unable to read the chart, visual acuity was evaluated using the finger counting method at 3 meters. Best-corrected visual acuity was determined using pinhole and refractive correction where applicable. Intraocular pressure was measured with a Goldmann applanation tonometer. Anterior segment evaluation was carried out using a slit-lamp biomicroscope (Nidek, Gamagori-shi, Japan). Pupils were dilated using tropicamide (0.8%) and phenylephrine (5%) eye drops to allow for posterior segment examination. Fundus evaluation was performed using a binocular indirect ophthalmoscope (Heine, Gilching, Germany) with +20D and +90D Volk lenses. Additional imaging included color fundus photography (Topcon, Tokyo, Japan) and optical coherence tomography of the macula (Nidek, Japan).

Laboratory investigations included hemoglobin estimation, glycated hemoglobin (HbA1c), lipid profile, and serum creatinine. All participants had active DFUs at the time of enrollment, and ulcer severity was graded using Wagner's classification [23], which is a standard, publicly available clinical tool for research use. According to Wagner's classification, grade 1 indicates superficial ulcers, grade 2 indicates ulcers extending to ligaments, tendons, joint capsules, or fascia without abscess or osteomyelitis, grade 3 indicates deep ulcers with abscess or osteomyelitis, grade 4 indicates gangrene localized to the forefoot, and grade 5 indicates extensive gangrene involving the entire foot.

Data collection and management

All relevant clinical and laboratory data were recorded in a predesigned data collection sheet. Personal identifiers were removed to maintain confidentiality, and each participant was assigned a unique study code.

Data analysis

Data were analyzed using SPSS version 23.0 for Windows (SPSS Inc., Chicago, Illinois, USA). Continuous variables were expressed as means with standard deviations, while categorical variables were presented as frequencies and percentages. Associations between categorical variables were examined using the chi-square test, with statistical significance set at a p-value of less than 0.05.

Ethical considerations

The study protocol received ethical approval from the Ethical Review Committee of Sher-E-Bangla Medical College Hospital (Ref No: SBMC/ECC/1102). All participants were informed about the scope and limitations of the study in their native language before enrollment. Written informed consent was obtained from each participant. Confidentiality of personal information was strictly maintained throughout the study. The study procedures did not interfere with participants' ongoing medical treatment, and no physical, mental, financial, or social harm was caused to any participant. Any complications arising during the study were managed promptly within the hospital setting.

## Results

This cross-sectional study was conducted from August 2021 to July 2022 at Sher-E-Bangla Medical College Hospital. We enrolled a total of 100 patients with T2DM who had a mean age of 55.7 ± 10.9 years. Most patients were in the 51-60 years age group (33 patients, 33%), followed by those aged >60 years (27 patients, 27%), 41-50 years (31 patients, 31%), and 31-40 years (9 patients, 9%). There was a male predominance (67 patients, 67%), while females accounted for 33 patients (33%). Regarding diabetes duration, 37 (37%) patients had the disease for >15 years, 30 (30%) patients for 11-15 years, 25 (25%) patients for 5-10 years, and 8 (8%) patients for <5 years, with an overall mean duration of 14.2 ± 6.6 years. Mean BMI was 23.0 ± 3.9 kg/m², and blood pressure averaged 121.7 ± 17.8/79.2 ± 10.6 mmHg. Table 1 summarizes the baseline demographic and clinical characteristics of the study population.

**Table 1:** Demographic and clinical characteristics of the study population (n=100)

Characteristics	n	%
Age (years), mean ± SD	55.7 ± 10.9	-
31-40	9	9
41-50	31	31
51-60	33	33
>60	27	27
Sex		
Male	67	67
Female	33	33
Duration of diabetes mellitus (years), mean ± SD	14.2 ± 6.6	-
<5	8	8
5-10	25	25
11-15	30	30
>15	37	37
Body mass index (kg/m²), mean ± SD	23.0 ± 3.9	-
Blood pressure (mmHg), mean ± SD		
Systolic blood pressure	121.7 ± 17.8	-
Diastolic blood pressure	79.2 ± 10.6	-

Smoking history was reported by 31 (31%) patients, while 69 (69%) patients were non-smokers. Betel nut chewing was noted in 17 (17%) patients, with 83 (83%) patients denying its use. Family history of diabetes was positive in 26 (26%) patients, while 74 (74%) patients had no such history. Regarding glycemic control, 11 (11.0%) patients were on no antidiabetic medication at the time of presentation. Among the remaining 89 patients, 36 (36.0%) were on oral hypoglycemic agents (OHAs) only, and 53 (53.0%) were on insulin with or without OHAs (Table 2).

**Table 2:** Distribution of study patients according to history (n=100) DM, diabetes mellitus; OHA, oral hypoglycemic agent

Characteristics	n	%
Smoking		
Yes	31	31.0
No	69	69.0
Betel nut use		
Yes	17	17.0
No	83	83.0
Family history of diabetes mellitus		
Yes	26	26.0
No	74	74.0
Drug history for glycemic control		
No medication	11	11.0
OHAs only	36	36.0
Insulin (± OHAs)	53	53.0

Visual acuity evaluation using Snellen charts revealed significant impairment across both eyes. Visual impairment (≤6/60) was present in 33 (33%) patients in the right eye and 36 (36%) patients in the left eye (Table 3).

**Table 3:** Visual acuity of the study subjects (n=100) Visual acuity measured using Snellen charts at 6 meters

Visual Acuity	Right Eye		Left Eye	
	n	%	n	%
6/12	11	11.0	13	13.0
6/18	19	19.0	22	22.0
6/24	15	15.0	14	14.0
6/36	23	23.0	15	15.0
≤6/60	33	33.0	36	36.0

**Figure 2:**
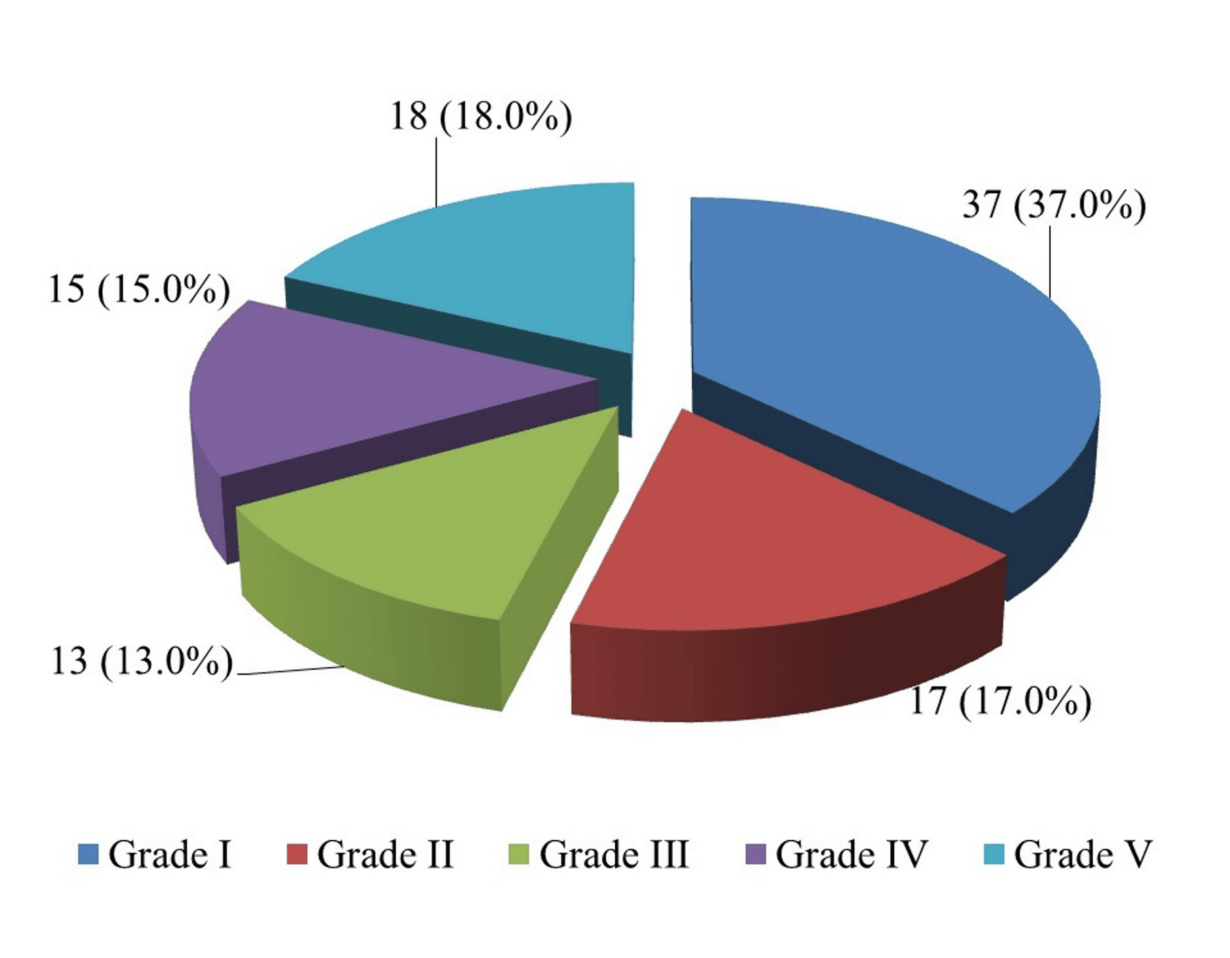
Distribution of diabetic foot ulcers using the Wagner grading system (n=100)

The majority of ulcers were grade I (37 patients, 37%), though advanced gangrenous lesions were also common, including grade V (18 patients, 18%), reflecting the tertiary referral nature of the cohort (Figure 2).

DR was identified in 80 (80%) patients using the Early Treatment Diabetic Retinopathy Study (ETDRS) classification. Among the study subjects, the distribution was as follows: no DR in 20 (20%) patients, mild non-proliferative diabetic retinopathy (NPDR) in 36 (36%) patients, moderate NPDR in 14 (14%) patients, severe NPDR in 21 (21%) patients, proliferative diabetic retinopathy (PDR) in 9 (9%) patients, high-risk PDR in 3 (3%) patients, and advanced diabetic eye disease (ADED) in 6 (6%) patients (Table 4).

**Table 4:** Distribution of diabetic retinopathy severity among study subjects DR, diabetic retinopathy; NPDR, non-proliferative diabetic retinopathy; PDR, proliferative diabetic retinopathy; ADED, advanced diabetic eye disease

Diabetic Retinopathy Category	n	%
No DR	20	20.0
Mild NPDR	36	36.0
Moderate NPDR	14	14.0
Severe NPDR	21	21.0
PDR	9	9.0
High-risk PDR	3	3.0
ADED	6	6.0

Patients with DR (n=80) had a significantly longer duration of diabetes compared to those without DR (n=20) (15.3 ± 6.6 vs. 9.7 ± 4.5 years; χ² = 7.52, df = 1, p = 0.006). Smoking was also more common in the DR group (31 patients, 38.8%, vs. 0 patients, 0%; χ² = 5.62, df = 1, p = 0.018). The difference in sex distribution between the two groups was not statistically significant (χ² = 0.72, df = 1, p = 0.395). No significant differences were also observed for age (t = 0.73, df = 98, p = 0.469), body mass index (t = -0.27, df = 98, p = 0.785), systolic blood pressure (t = 0.26, df = 98, p = 0.795), or diastolic blood pressure (t = 0.49, df = 98, p = 0.613) (Table 5).

**Table 5:** Comparison of characteristics between DFU patients with and without DR *A p-value of <0.05 is considered statistically significant DFU, diabetic foot ulcer; DR, diabetic retinopathy; BMI, body mass index; SBP, systolic blood pressure; DBP, Diastolic blood pressure

Characteristics	DFU with DR (n=80)	DFU without DR (n=20)	p-Value
Age (years), mean ± SD	56.1 ± 11.7	54.1 ± 7.6	0.469
Sex, male	52 (65.0%)	15 (75.0%)	0.395
BMI (kg/m²), mean ± SD	23.0 ± 3.8	23.2 ± 4.2	0.785
Duration of DM (years), mean ± SD	15.3 ± 6.6	9.7 ± 4.5	0.006*
Smoking	31 (38.8%)	0 (0%)	0.018*
SBP (mmHg), mean ± SD	122.2 ± 10.9	119.4 ± 32.6	0.795
DBP (mmHg), mean ± SD	79.8 ± 11.2	77.8 ± 8.3	0.613

Hemoglobin levels were significantly lower in the DR group (10.9 ± 1.8 vs. 12.1 ± 1.1 g/dL; t = -2.02, df = 98, p = 0.046). No significant differences were observed for glycated hemoglobin, serum creatinine, cholesterol, triglycerides, high-density lipoprotein, or low-density lipoprotein between the two groups (Table 6).

**Table 6:** Biochemical characteristics comparison between DFU patients with and without DR An independent t-test was used for all comparisons. *A p-value of <0.05 is considered statistically significant. DFU, diabetic foot ulcer; DR, diabetic retinopathy; HbA1c, glycated hemoglobin; HDL, high-density lipoprotein; LDL, low-density lipoprotein

Parameter	DFU with DR (n=80)	DFU without DR (n=20)	p-Value
Hemoglobin (g/dL), mean ± SD	10.9 ± 1.8	12.1 ± 1.1	0.046*
HbA1c (%), mean ± SD	10.8 ± 1.8	9.4 ± 1.7	0.698
Serum creatinine (mg/dL), mean ± SD	1.5 ± 0.3	1.6 ± 0.4	0.270
Cholesterol (mg/dL), mean ± SD	151.4 ± 41.3	152.3 ± 35.8	0.874
Triglycerides (mg/dL), mean ± SD	135.6 ± 97.4	136.9 ± 65.7	0.892
HDL (mg/dL), mean ± SD	37.8 ± 11.5	43.2 ± 10.9	0.072
LDL (mg/dL), mean ± SD	81.0 ± 32.0	82.3 ± 33.4	0.846

Table 7 presents the chi-square test statistics for the association between DR severity and Wagner grade of DFU. A significant overall association was found between the severity of DR and higher Wagner grades (χ² = 42.18, df = 16, p = 0.001). Mild NPDR was most common in patients with Wagner grade I ulcers (19 patients, 51.4%), while severe NPDR was predominantly observed in patients with Wagner grade IV (7 patients, 46.7%) and grade V (11 patients, 61.1%) ulcers (χ² = 48.56, df = 16, p = 0.001). PDR was observed only in patients with Wagner grade IV (3 patients, 20.0%) and grade V (6 patients, 33.3%) ulcers (χ² = 22.14, df = 16, p = 0.034). No significant associations were observed for moderate NPDR (χ² = 14.28, df = 16, p = 0.183), high-risk PDR (χ² = 12.06, df = 16, p = 0.059), or advanced diabetic eye disease (χ² = 14.98, df = 16, p = 0.128).

**Table 7:** Association between severity of DR and Wagner grade of DFU A chi-square test was used for all comparisons. *A p-value of <0.05 was considered statistically significant. DR, diabetic retinopathy; NPDR, non-proliferative diabetic retinopathy; PDR, proliferative diabetic retinopathy; ADED, advanced diabetic eye disease

Diabetic Retinopathy Category	Grade I (n=37)	Grade II (n=17)	Grade III (n=13)	Grade IV (n=15)	Grade V (n=18)	χ²	p-Value
	n (%)	n (%)	n (%)	n (%)	n (%)		
No diabetic retinopathy	18 (48.6)	2 (11.8)	0 (0)	0 (0)	0 (0)	42.18	0.001*
Mild NPDR	19 (51.4)	9 (52.9)	5 (38.5)	3 (20.0)	0 (0)	35.42	0.001*
Moderate NPDR	0 (0)	6 (35.3)	5 (38.5)	2 (13.3)	1 (5.6)	14.28	0.183
Severe NPDR	0 (0)	0 (0)	3 (23.1)	7 (46.7)	11 (61.1)	48.56	0.001*
PDR	0 (0)	0 (0)	0 (0)	3 (20.0)	6 (33.3)	22.14	0.034*
High-risk PDR	0 (0)	0 (0)	0 (0)	2 (13.3)	1 (5.6)	12.06	0.059
ADED	0 (0)	0 (0)	0 (0)	2 (13.3)	4 (22.2)	14.98	0.128

## Discussion

DR and DFUs are two of the most debilitating complications of T2DM and commonly co-exist as signs of advanced systemic vascular injury. The present cross-sectional study, performed in a tertiary care hospital, Barishal, Bangladesh, found an alarmingly high prevalence of DR (80%) among 100 patients with T2DM and active DFU, with progression of severity of retinopathy parallel with the Wagner ulcer grades. These findings highlight the association between DR and DFU as manifestations of systemic microvascular disease and the imperative for a joint screening and management approach in resource-limited settings. The demographic profile of the group of participants is similar to that in other similar groups. The mean ages were 56.1 ± 11.7 years and 54.1 ± 7.6 years in the DR-positive and DR-negative groups, respectively, indicating no statistically significant difference (p = 0.469). This is generally consistent with findings from other regions of South Asia and elsewhere, which show that patients with concomitant DR and DFU tend to present in their mid-50s [24,25]. However, more recently, it appears that age might not be the most important factor and that the cumulative exposure to hyperglycemia is more important [19,26]. Males were more likely in both groups (65 vs. 75%), which is consistent with the general referral biases seen in many LMICs, in which men may present after a longer duration of disease or due to their occupation [20,27]. Such gender patterns need to be further discussed in studies that place them in the context of their culture.

A particularly worthy finding was the significantly longer mean duration of diabetes in patients with DR (15.29 ± 6.64 years) as compared to those without (9.67 ± 4.50 years; p = 0.006). This is a strong association in accordance with a number of previous studies and is consistent with the notion that sustained hyperglycemia is responsible for parallel progression of damage to the microvasculature in the retina and lower extremities [20,24,28]. Recent evidence indicates that patients with DR have a significantly elevated risk for DFU (e.g., OR 1.74, 95% CI 1.36-2.23), presumably due to common endothelial dysfunction, basement membrane thickening, and pericyte loss [26,28].

In the present study, smoking was also only found in the DR group (38.8% vs. 0%; p = 0.018), which is consistent with the increased data showing tobacco use being associated with the accelerated vascular injury and impaired wound healing associated with diabetes [29]. Lower hemoglobin levels in the DR-positive group (10.9 ± 1.8 vs. 12.1 ± 1.1 g/dL; p = 0.046) further suggest the possibility of anemia, which can be seen to be related to chronic inflammatory process, renal impairment, or nutritional deficits among the LMIC populations and may contribute to increased tissue ischemia both in the retina and the foot. Blood pressure, BMI, HbA1c, lipid profile, and serum creatinine were not significantly different between the groups in this cohort and were slightly different from studies where poorer glycemic control or dyslipidemia was associated more strongly [20]. This discrepancy is perhaps a reflection of the degree of disease present at presentation in a tertiary referral center in which the majority of patients have long-standing hyperglycemia (mean overall duration 14.2 ± 6.6 years) and multiple complications. In such populations, the duration of diabetes and cumulative microvascular insult may be more important determinants than acute glycemic markers measured at a single time point.

The distribution of DR severity was notable. Mild NPDR was the most common finding (36%). However, more severe forms were also well represented: severe NPDR (21%), PDR (9%), high-risk PDR (3%), and advanced diabetic eye disease (6%). Additionally, nearly one-third of the sample (33%-36%) had a visual acuity of 6/60 or worse. Crucially, a significant association was observed between the severity of DR and the Wagner grade of DFUs (p < 0.05). Mild NPDR was most common in grade I ulcers (51.4%), and severe NPDR and PDR were significantly more common in grades IV-V (up to 61.1% severe NPDR and 33.3% PDR in grade V). This dose-response relationship is in line with the recent prospective data and meta-analysis, which show that the higher the Wagner grade, the more advanced the DR and vice versa [18,26].

These patterns are most likely to be due to some common underlying mechanisms: oxidative stress, AGE formation, and inflammatory cytokines resulting from chronic hyperglycemia damage the microvasculature of the retina and simultaneously promote peripheral neuropathy, ischemia, and poor wound healing in the lower extremities [5,16]. The presence of one of the advanced microvascular complications is therefore a sentinel clinical sign for the other. In Bangladesh, despite a significantly increasing prevalence of diabetes (around 13% among adults) and a high percentage of undiagnosed and uncontrolled diabetes, delayed diagnosis and scattered care exacerbate this two-way risk [22].

The high co-occurrence rate revealed herein (80% DR in DFU patients) is higher than many general populations of people with diabetes but much in line with tertiary center populations in South Asia and elsewhere, where patients tend to have a late presentation with multiple complications [30]. This adds weight to the arguments for routine ophthalmic evaluation in all DFU admissions, as recommended by international guidelines as well as new research from LMICs [18]. Integrated "eye-foot" clinics could be used to promote early detection of vision-threatening DR and prevention of blindness and optimize foot care to minimize the risk of amputation. Patient education that includes foot hygiene, glycemic control, smoking cessation, and regular screening are equally important especially in areas that have cultural practices (such as the use of betel nuts) and socioeconomic barriers that delay the onset of care. Methodological strengths of this study are extensive ophthalmic evaluation using ETDRS grading, color fundus photography and optical coherence tomography, and standardized Wagner's classification.

However, limitations such as the small sample size, the purposeful sampling, the single-center design, the short duration of the study, and the failure to measure microalbuminuria limit the generalizability of the results and exclude the ability to make causal inferences. The COVID-19 pandemic further worsened the limited enrollment, indicating logistic difficulties in research in LMICs. The uneven distribution across some DR and Wagner subcategories limits the precision of subgroup analyses, though the overall association remained statistically significant.

## Conclusions

This study demonstrates that DR is highly prevalent and frequently severe among patients with DFUs in Bangladesh. The severity of retinopathy closely parallels the grade of foot ulceration and is significantly associated with longer duration of diabetes, smoking, and lower hemoglobin levels. These findings support the concept that DR and DFU are concurrent manifestations of systemic microvascular and metabolic dysfunction. Importantly, this study highlights that the presence of DFU should be recognized as a clinical indicator of an increased risk for vision-threatening DR. Routine ophthalmologic screening should therefore be considered standard care in patients presenting with DFU to enable early detection and timely intervention. The strong association observed between DR and DFUs underscores the need for integrated, multidisciplinary, and proactive management strategies. Further large-scale, multicenter prospective studies in South Asia are warranted to validate these findings and better understand long-term outcomes and the impact of coordinated interventions. Strengthening early screening programs, optimizing glycemic control, and improving patient education may significantly reduce the dual burden of blindness and limb loss in resource-limited settings.
